# Impact of acute stress on gastric interstitial cells of Cajal disorder

**DOI:** 10.3389/fcell.2026.1872384

**Published:** 2026-08-26

**Authors:** Zhen-peng Huang, Ke Wang, Hu Qiu, Ji-wei Wan, Zhi Yang, Ling-wang Kong, Zhuo-yuan Li

**Affiliations:** 1 Faculty of Nursing, Guangxi University of Chinese Medicine, Nanning, China; 2 College of Clinical Medicine, Xi’an Medical University, Xi’an, China; 3 Department of Respiratory and Critical Care Medicine, The First Affiliated Hospital of Xi’an Jiaotong University, Xi’an, China; 4 Department of Oncology, Renmin Hospital of Wuhan University, Wuhan, China; 5 School of Public Health, Xi’an Medical University, Xi’an, China

**Keywords:** acute stress, inflammation, interstitial cells of Cajal, oxidative stress, protein 53, SCF/c-kit pathway, Wnt/β-catenin pathway

## Abstract

**Introduction:**

Acute stress can disrupt gastrointestinal function. Interstitial cells of Cajal (ICCs) regulate gastric motility; however, the effects underlying their alterations during stress remain unclear. We aimed to explore the potential impact of acute stress on alterations in gastric ICCs.

**Methods:**

Animals were assigned to one control and four study groups (0, 24, and 72 h). Acute stress was induced via right chest puncture using a Hopkinson bar. Lung injury was assessed using hematoxylin-eosin staining, gastric ICCs were examined using immunohistochemistry, immunohistofluorescence, and TdT-mediated dUTP nick-end labeling assays. Transmission electron microscopy was used to evaluate ultrastructural changes. Western blotting and real-time polymerase chain reaction were used to analyze SCF/c-kit, protein 53 (p53), and the Wnt/β-catenin signaling pathways. Enzyme-linked immunosorbent assay was used to quantify nuclear factor kappa B (NF-κB) p65, interleukin (IL)-1β, IL-6, IL-9, IL-10, tumor necrosis factor-alpha (TNF-α), and cortisol. Spectrophotometry was used to determine the levels of reactive oxygen species (ROS) and malondialdehyde (MDA).

**Results:**

The lung tissue exhibited congestion, edema, and the destruction of the alveolar structure following puncture. The gastric ICCs tested positive for the surface markers CD117/c-kit and CD34. The population and apoptosis of the gastric ICCs were altered in the acute stress condition. Moreover, acute stress caused dysregulated SCF/c-kit, Wnt/β-catenin, p53 and NF-κB pathways. IL-1β, IL-6, IL-9, IL-10, TNF-α, and cortisol demonstrated altered expression levels, whereas ROS and MDA were upregulated in the acute stress condition.

**Conclusion:**

Acute stress damages gastric ICCs through the Wnt/β-catenin, JAK/STAT, and NF-κB signaling pathways, as well as p53-and SCF/c-kit pathway-mediated abnormalities and oxidative stress, contributing to functional alterations in gastric ICCs.

## Introduction

Traffic accident is a critical challenge in public health globally. It is associated with over 1.25 million deaths annually worldwide and exhibits an increasing trend (Hua et al., 2023; Atreya et al., 2021; Foroutaghe et al., 2019). Thoracic trauma is a common and lethal injury in traffic accidents (Foroutaghe et al., 2019). Experience of traffic accidents induces physiological and psychological acute stress conditions (Foroutaghe et al., 2019; Pozzato et al., 2021). Psychological and physical acute stressors significantly contribute to gastrointestinal dysfunction, particularly gastrointestinal dysmotility (Schaper and Stengel, 2022; Boeckxstaens et al., 2016). In addition, acute stress markedly influences the interaction between the gastrointestinal tract and central nervous system and affect gut motility (Boeckxstaens et al., 2016; Bagues et al., 2021).

The interstitial cells of Cajal (ICCs) are in gastric tissues and function as smooth muscle pacemakers in the digestive tract, promoting gastrointestinal slow wave and regulating gastrointestinal tract neurotransmitters (Sanders et al., 2023; Sweet et al., 2024). Recent findings suggest that ICCs participate in gastrointestinal motility disorders such as gastric dysmotility such as functional dyspepsia and gastroparesis (Wang T. H. et al., 2021; Zeng et al., 2024; Moraveji et al., 2016). However, changes in gastric ICCs and the potential mechanisms during acute stress remain unclear. In this study, we aimed to explore the cellular and molecular mechanisms of gastric ICC damage in the acute stress condition.

## Materials and methods

### Animals and animal experiments

We obtained 24 New Zealand rabbits, including males and females (weight: 2.0–2.5 kg) from the medical laboratory animal center of Xi’an Jiao Tong University. All rabbits were maintained in standard laboratory conditions (22 °C ± 2 °C; relative humidity, 40%–60%) with a 12-h light/dark cycle. The rabbits were given food and water *ad libitum*. All animal experiments were conducted in accordance with the ARRIVE guidelines 2.0 and were approved by the institutional medical ethics committee of Xi’an Medical University (Approval No.: XYLS2021005).

Rabbits were randomly divided into four groups, including one healthy control group and three study groups. The study groups included the 0-, 24-, and 72-h groups. There were six animals in each group. Following anesthesia using 1 mL/kg 3% pentobarbital sodium (New Asia Pharmaceutical, Shanghai, China) via ear-vein injection, the rabbits in the study groups were subjected to a right chest puncture using a Hopkinson bar with an acceleration of 2,600 *g*. The lungs and gastric tissues of the animals in the 0-h group were harvested immediately after the puncture. Following anesthesia, lung and tissue harvests were performed on the animals in the 24- and 72-h groups at 24 and 72 h, respectively, after puncture.

### Hematoxylin-eosin staining

The lung tissues from the healthy control and 0-h groups were fixed in a 4% polyformaldehyde solution and embedded in paraffin. The paraffin-embedded lung tissues were sectioned into 5-μm-thick slices and mounted on positively-charged slides. After staining the sections with Harris’ hematoxylin for 5 min, they were rinsed under running water and differentiated with a 1% hydrochloric acid solution for 5–10 s. After another gentle rinse with running water, the tissues were re-stained with 0.5% eosin for 20 min and further stained for 30–60 s.

### Immunohistochemistry staining

For immunohistochemical evaluation, paraffinized sections were subjected to antigen retrieval using a microwave-based technique. Gastric tissues from the healthy control group were fixed in 4% paraformaldehyde, embedded in paraffin, and sectioned at a thickness of 5 µm onto positively-charged slides. Subsequently, these sections were probed with primary antibodies, including a rabbit anti-c-kit polyclonal antibody against CD117/c-kit (Bioss, Beijing, China) and a rabbit anti-CD34 polyclonal antibody against CD34 (Bioss, Beijing, China). Following a 24-h incubation at 22 °C and subsequent application of appropriate secondary antibodies, staining was completed using diaminobenzidine as the chromogen, and hematoxylin was used for counterstaining.

### Immunohistofluorescence and TdT-mediated dUTP nick-end labeling (TUNEL)

Gastric tissues from all groups were fixed in 4% paraformaldehyde and stored for 30 min at 4 °C. After three 10-min washes with 1× phosphate-buffered saline (PBS) the specimens were sectioned. The sections were permeabilized with PBS containing 0.3% Triton X-100, blocked with normal goat serum for 30 min at 22 °C, and incubated overnight at 4 °C with primary antibodies, including rabbit anti-c-kit (Bioss, Beijing, China) and anti-CD34 (Bioss, Beijing, China) polyclonal antibodies. Following three 10-min washes with 1× Tris buffered saline with Tween-20 (TBST), the sections were incubated with a CY3-conjugated secondary antibody for 1 h at 22 °C. Unbound secondary antibody was removed through three 5-min washes with 1× TBST.

Gastric ICC death was assessed across all groups using a TUNEL assay (*In Situ* Cell Death Detection Kit, Fluorescein; Roche Applied Science, Mannheim, Germany), strictly following the manufacturer’s protocol.

### Transmission electron microscopy

We collected 5 × 5 mm gastric tissue blocks from all animal groups and soaked them in a 2.5% glutaraldehyde solution for 12 h at 4 °C. The specimens were subjected to three 15-min washes with 1× PBS solution. The gastric specimens of each group were immersed in 1% osmium acid solution, shaken, and stained for 2 h at 4 °C. Subsequently, all specimens were placed in a 2% acetic acid dioxyuranium solution and shaken at 22 °C for staining for 1 h. Tissue dehydration was conducted using an ethanol solution. The gastric specimens from each group were dipped into pure acetone for 15 min three times. Epone 812 solution was added to embed the gastric specimens, which were stored for 12 h at 37 °C. Next, the specimens were transferred to a 60 °C oven for drying for 48 h. Tissue ultrathin sectioning was performed using a pathological sectioning machine, with each section having a 50-nm thickness. Ultrathin tissue sections were stained with alcohol-acetate dioxyuranium and lead citrate. The sections were imaged using a transmission electron microscope (HT7800, Hitachi, Tokyo, Japan).

### Protein extraction and western blotting

Proteins were extracted from 150 mg of gastric tissue per group using a radioimmunoprecipitation assay lysis buffer. The proteins were separated via electrophoresis on 10% sodium dodecyl sulfate-polyacrylamide gels and transferred onto nitrocellulose membranes (Pierce Biotechnology, Inc., Rockford, IL, USA). After blocking with 5% skim milk in 1× TBST for 2 h at 22 °C, the membranes were incubated overnight at 4 °C with primary antibodies anti-c-kit antibody (Bioss, Beijing, China), anti-SCF antibody (Abcam, Cambridge, UK), Wnt3a rabbit primary antibody (Bioss, Beijing, China), or β-catenin rabbit primary antibody (Bioss, Beijing, China). Following washes, the membranes were incubated with horseradish peroxidase-conjugated secondary antibodies for 1 h at 22 °C. Protein bands were visualized via chemiluminescence (ECL; Amersham, Pittsburgh, PA, USA) using an X-ray film (Kadok China Investment Co. Ltd., Xiamen, China), and their optical density was quantified with an Alpha Innotech system (Alpha Innotech Co., San Leandro, CA, USA).

### RNA extraction and real-time polymerase chain reaction

Total RNA was isolated from 150 mg of gastric muscularis tissue using a TRIzol reagent (Invitrogen, Carlsbad, CA, USA). cDNA was synthesized from 4.38 µg of total RNA and amplified for 40 cycles under the following conditions: denaturation at 50 °C for 2 min and 95 °C for 10 min, annealing at 95 °C for 30 s, and extension at 60 °C for 30 s. Primer sequences for β-actin, p53, JAK-1, and STAT-1 are listed in Table 1. Polymerase chain reaction products were analyzed using electrophoresis on a 1.5% agarose gel. Gene expression was normalized to β-actin, and relative mRNA levels were calculated from the ratio of target gene to β-actin amplification products.

**TABLE 1:** The primers for β-actin, JAK-1, STAT-1 and p53.

Name	Primer	Sequence	Size
Rabbit β-actin	Forward	GTGGACATCCGCAAGGAC	200bp
	Reverse	TGGAAGGTGGAGAGCGAG	
Rabbit JAK-1	Forward	GCC​TGG​CTC​CTA​TTC​GAG​AC	211bp
	Forward	GCC​TGG​CTC​CTA​TTC​GAG​AC	
Rabbit STAT-1	Forward	GTG​ATC​TCC​AAC​GTC​AGC​CA	267bp
	Reverse	TTC​CTT​GCA​GAA​CCT​CGT​CC	
Rabbit p53	Forward	GTT​CGA​GAT​GTT​CCG​AGA​GC	165bp
	Reverse	GAG​TCA​GGC​CCC​TCT​CTC​TT	

### Enzyme-linked immunosorbent assay (ELISA)

We collected 150 mg of gastric tissue blocks from all animal groups, sectioned the tissue blocks, and ground them in an ice bath. Subsequently, the homogenate was centrifuged at 3,000 rpm for 10 min, and the supernatant was collected. Nuclear factor kappa B (NF-κB) p65 (Meimian, Yancheng, China), interleukin (IL)-1β (Meimian, Yancheng, China), IL-6 (Jonlnbio, Shanghai, China), IL-9 (Jonlnbio, Shanghai, China), IL-10 (Jonlnbio, Shanghai, China), tumor necrosis factor-alpha (TNF)-α (Jonlnbio, Shanghai, China), and cortisol (Jonlnbio, Shanghai, China) ELISA kits were used to measure their respective expression levels in the collected gastric tissues. ELISA procedures were performed according to the manufacturer’s instructions. The absorbance of each well was measured at 450 nm using a microplate reader (Thermo Scientific Multiskan FC, Waltham, USA).

### Spectrophotometry

We collected 150 mg of gastric tissue blocks from all animal groups, sectioned the tissue blocks, and ground them in an ice bath. Subsequently, the homogenate was centrifuged at 3,000 rpm for 10 min, and the supernatant was collected. The expression levels of reactive oxygen species (ROS) and malondialdehyde (MDA) were evaluated using ROS (Njjcbio, Nanjing, China) and MDA (Njjcbio, Nanjing, China) detection assays, respectively. ROS and MDA detection procedures were performed according to the manufacturer’s instructions. The absorbance of each well was measured at 532 nm on a microplate reader (Thermo Scientific Multiskan FC, Waltham, USA) to detect MDA. To determine the ROS levels, the absorbance of each well was measured at optimal excitation and emission wavelengths of 488 and 525 nm, respectively, using a microplate reader (Thermo Scientific Multiskan FC, Waltham, USA).

### Statistical analysis

All statistical analyses were performed using SPSS for Windows (Version 27.0; SPSS, Chicago, IL, USA). Continuous variables are presented as means ± standard deviation and were compared using analysis of variance. A two-sided *P*-value of <0.05 was considered statistically significant.

## Results

### Evaluation of lung injury

**FIGURE 1:**
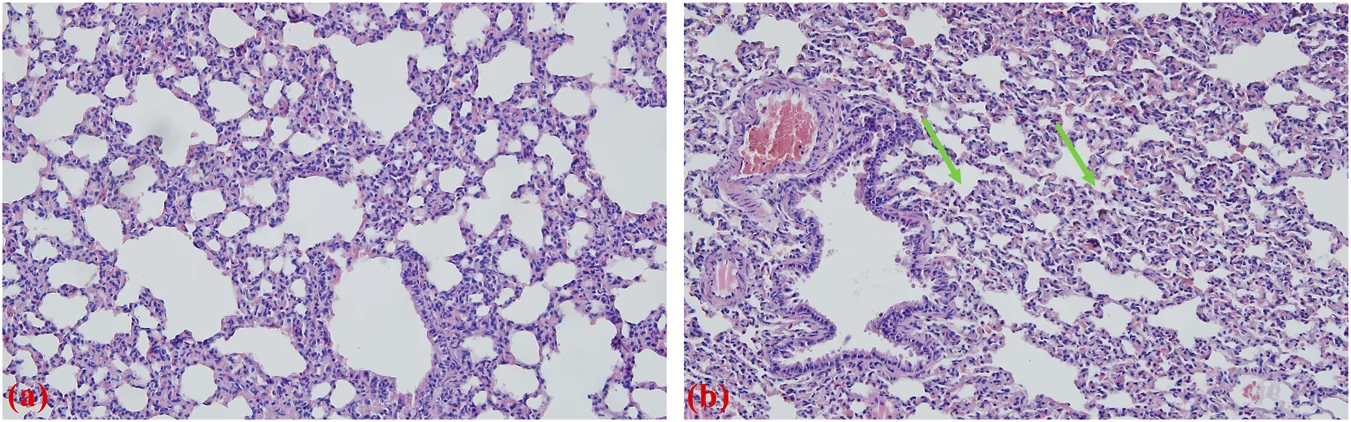
Hematoxylin-eosin staining on lung tissue (200×). Lung tissue was complete in the healthy control group, without evident inflammatory cell infiltration **(a)**. In the study group, the lung tissue indicated the permeation of red blood cells into the alveolar wall interstitium or around the bronchi, as well as extensive cellular infiltration into the alveolar space, alveolar obliteration, increased lung consolidation, and expanded alveolar interstitium (Shown in green arrows) **(b)**.

In this study, all animals survived before and after puncture using the Hopkinson bar. In the macro-pathological examination of all animals, pulmonary congestion and edema were observed in all study groups, whereas no obvious pathological changes were observed in the healthy control group. Histopathological staining indicated that the lung tissue was complete in the healthy control group, without evident inflammatory cell infiltration. However, in the study groups, the lung tissue indicated the permeation of red blood cells into the alveolar wall interstitium or around the bronchi, as well as extensive cellular infiltration into the alveolar space, alveolar obliteration, increased lung consolidation, and expanded alveolar interstitium (Figure 1).

### Immunohistochemical observation on gastric ICCs

**FIGURE 2:**
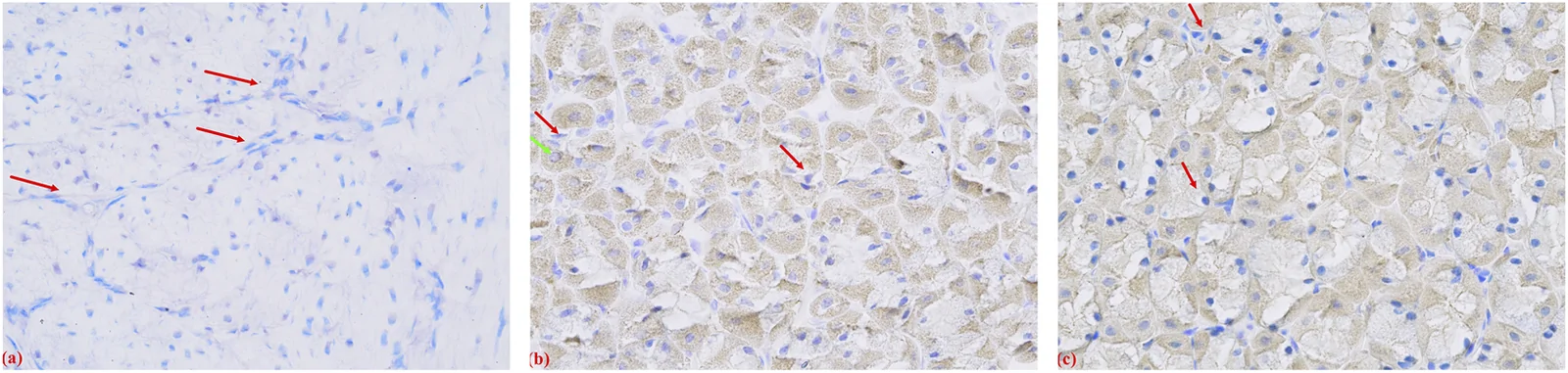
Immunohistochemical observation on gastric ICCs (400×). Gastric ICCs (red arrows) exhibited a predominantly fusiform shape with slender lateral branches and were primarily within the muscular layer, aligned parallel to smooth muscle cells in the muscularis propria **(a)**. ICCs tested positive for the surface markers CD117/c-kit **(b)** and CD34 **(c)**. Each ICC possessed two to five elongated processes, forming an interconnected network throughout the gastric wall. Gastric ICCs were individually or in small clusters of two to three cells. Mast cells were occasionally around gastric ICCs in single, large, and round and tested positive for CD117/c-kit (green arrow).

We used methylene blue to stain the gastric tissue and used anti-CD117/c-kit and anti-CD34 antibodies to assess gastric ICCs via immunohistochemistry. These cells exhibited a predominantly fusiform shape with slender lateral branches and were primarily within the muscular layer, often aligned parallel to smooth muscle cells in the muscularis propria. ICCs tested positive for the surface markers CD117/c-kit and CD34. Each ICC possessed two to five elongated processes, forming an interconnected network throughout the gastric wall. Gastric ICCs were observed individually or in small clusters of two to 3 cells. Mast cells were occasionally around gastric ICCs and tested positive for CD117/c-kit; however, they were single, large, and round, differentiating them from ICCs (Figure 2).

### Alterations in the population and apoptosis rate of gastric ICCs in acute stress

**FIGURE 3:**
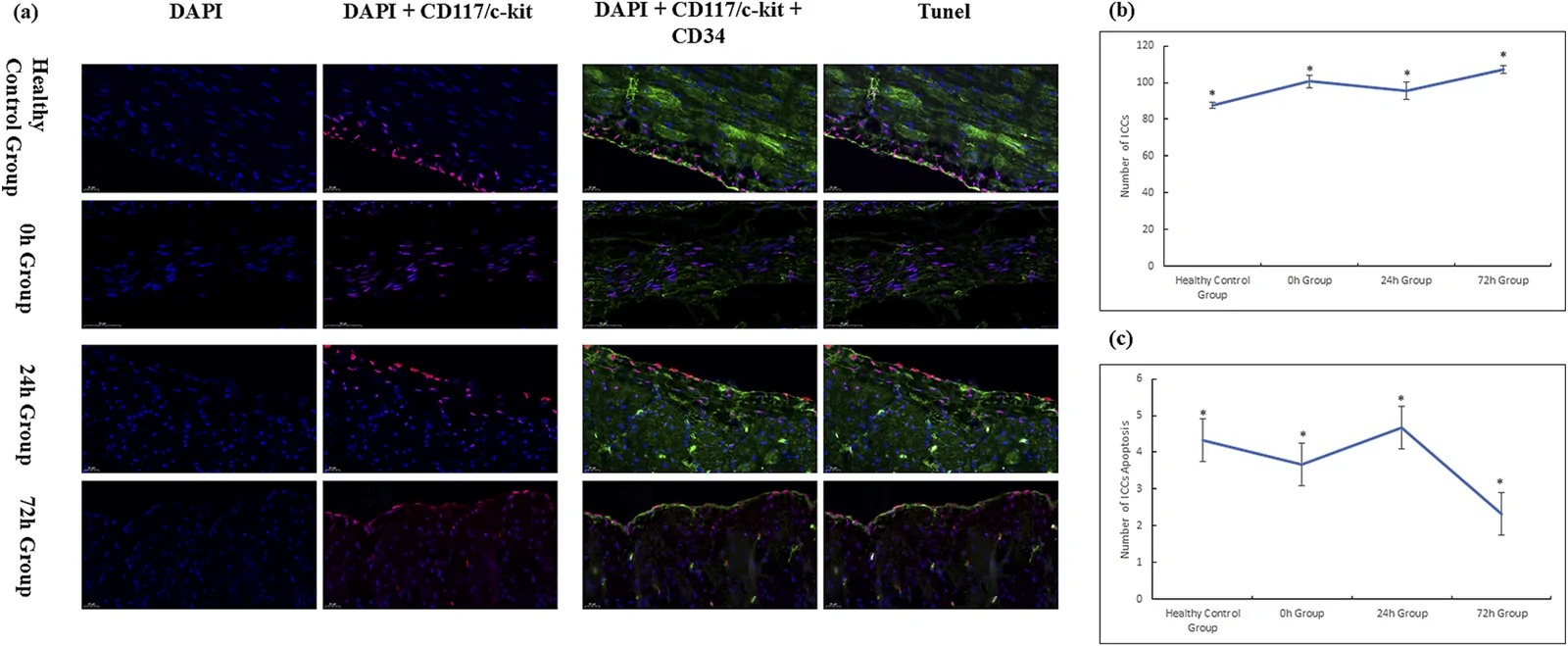
Numbers and apoptosis rate of gastric ICCs in acute stress. Taking immunohistofluorescence observation in 400× **(a)**. The population of gastric ICCs increased slightly immediately after puncture, followed by a decrease and an increase at 24 and 72 h after puncture, respectively (P< 0.05) **(b)**. The apoptosis rate of gastric ICCs decreased slightly immediately after puncture, followed by an increase and a decrease at 24 and 72 h after puncture, respectively (P < 0.05) **(c)**.

Three 400✕ magnification fields from the healthy control and all study groups were randomly selected for the immunofluorescence observation and TUNEL detection; the number of gastric ICCs changed significantly during acute stress. In addition, acute stress altered the death rates of gastric ICCs. The population of gastric ICCs increased slightly immediately after puncture, followed by a decrease and an increase at 24 and 72 h after puncture, respectively. Similarly, the apoptosis rate of gastric ICCs decreased slightly immediately after puncture, followed by an increase and a decrease at 24 and 72 h after puncture, respectively (all P < 0.05) (Figure 3).

### Morphological changes in gastric ICCs

**FIGURE 4:**
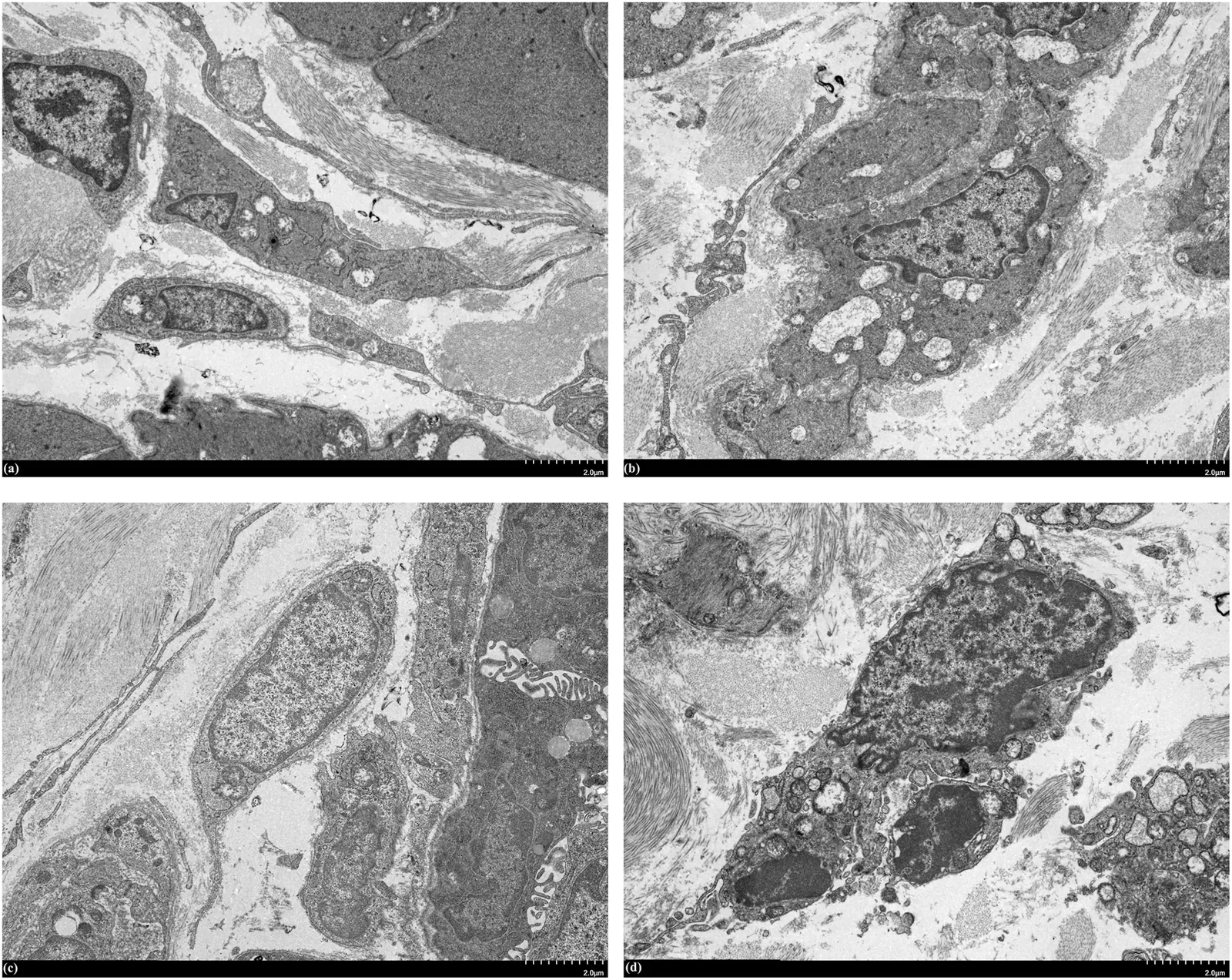
Transmission electron microscopy observation on gastric ICCs (1600×). Gastric ICCs were distributed in the subepithelial connective tissue layer and muscularis propria. Gastric ICCs were fusiform or astrocyte, had abundant cytoplasm, and their cell nucleus were big and round **(a)**. Gastric ICCs were connected by synapses, and the number of connected cells was increased immediately after puncture **(b)**; however, the population of the connected cells decreased at 24 h after puncture **(c)**. Subsequently, the cell connection recovered, and the net-like structure was more compact at 72 h after puncture **(d)**.

Transmission electron microscopy revealed that gastric ICCs were distributed in the subepithelial connective tissue layer and muscularis propria. Gastric ICCs were fusiform or astrocyte, had abundant cytoplasm, and their cell nucleus were big and round. Gastric ICCs were connected by synapses, and the number of connected cells was increased immediately after puncture; however, the population of the connected cells decreased at 24 h after puncture. Subsequently, the cell connection recovered, and the net-like structure was more compact at 72 h after puncture (Figure 4).

### Changes in the expression of the SCF/c-kit signaling pathway

**FIGURE 5:**
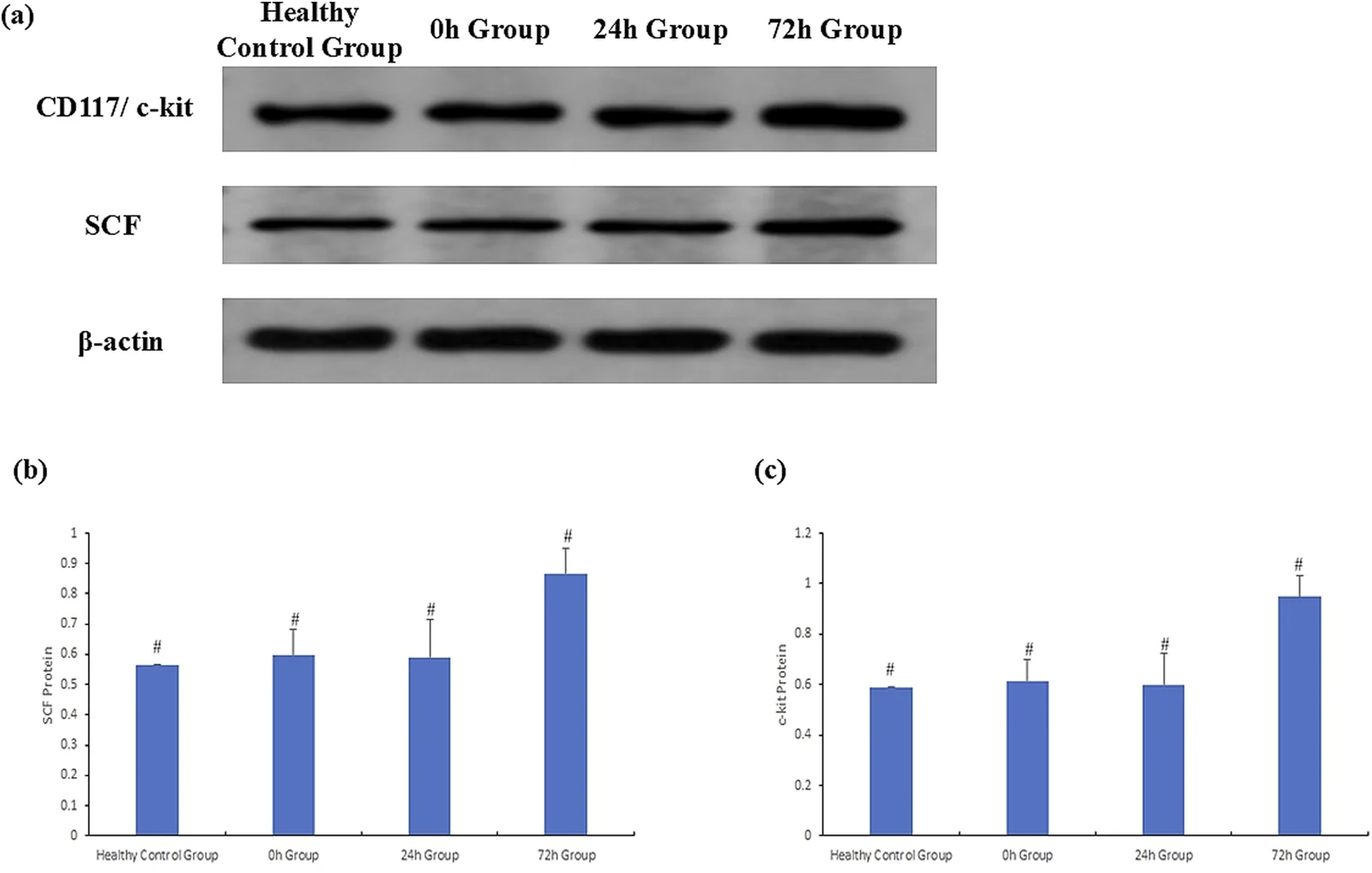
Changes in the expression of the SCF/c-kit signaling pathway. The SCF/c-kit signaling pathway was slightly upregulated immediately after puncture, followed by downregulation and upregulation at 24 and 72 h after puncture, respectively (all P < 0.05) **(a–c)**.

Western blotting indicated that the protein expression levels of CD117/c-kit and SCF were altered during acute stress. The SCF/c-kit signaling pathway was slightly upregulated immediately after puncture, followed by downregulation and upregulation at 24 and 72 h, respectively after puncture (all P < 0.05) (Figure 5).

### Changes in the expression of the Wnt/β-catenin signaling pathway

**FIGURE 6:**
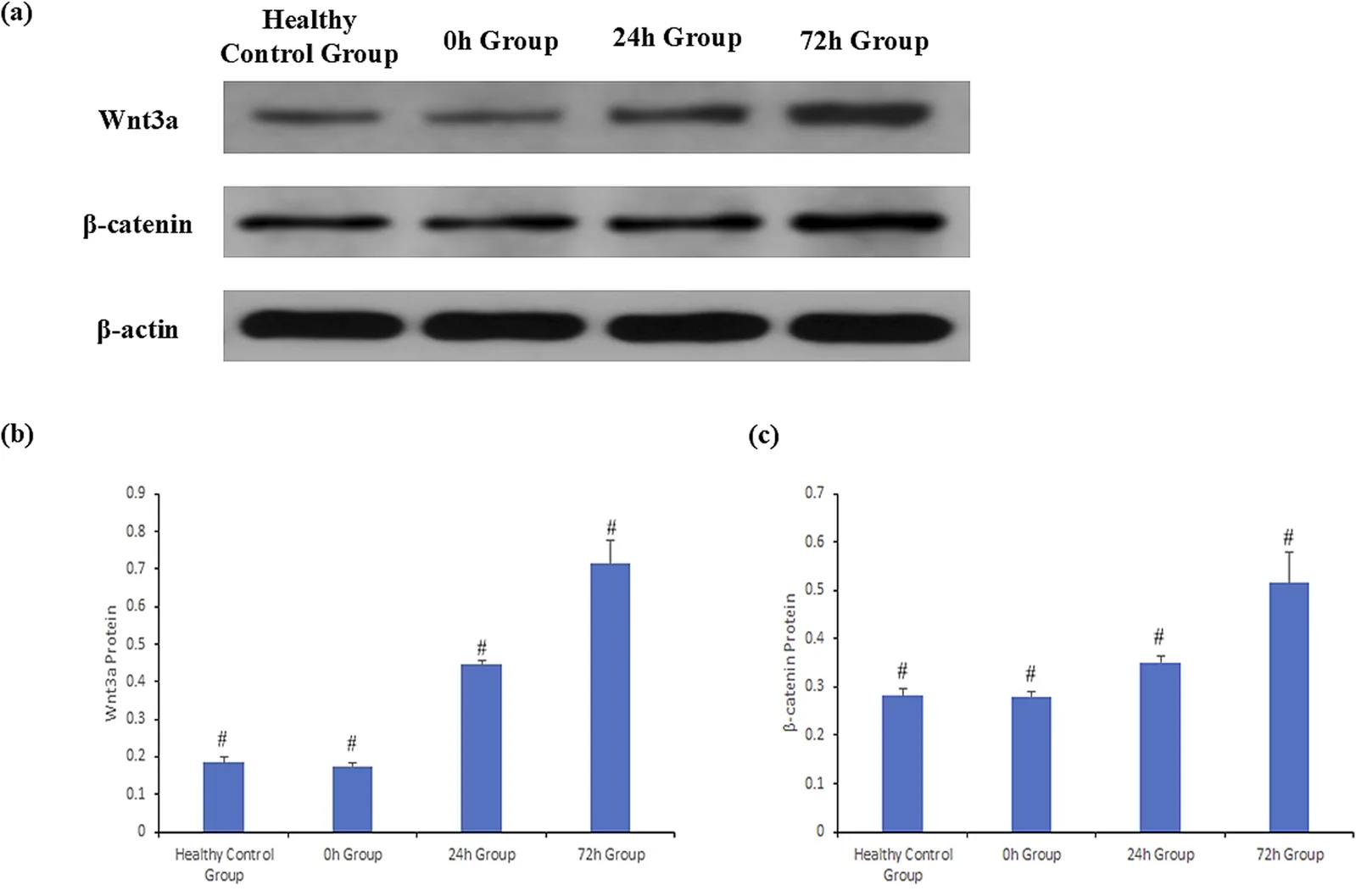
Changes in the expression of the Wnt/β-catenin signaling pathway. The Wnt/β-catenin signaling pathway was slightly upregulated immediately after puncture, followed by downregulation and upregulation at 24 and 72 h after puncture, respectively (all P < 0.05) **(a-c)**.

Furthermore, Western blotting indicated that the protein expression levels of Wnt 3a and β-catenin were altered in acute stress. The Wnt/β-catenin signaling pathway was slightly upregulated immediately after puncture. However, at 24 h after puncture, the pathway was downregulated; upregulation was restored at 72 h after puncture (all P < 0.05) (Figure 6).

### Changes in the expression of the p53 signaling pathway

**FIGURE 7:**
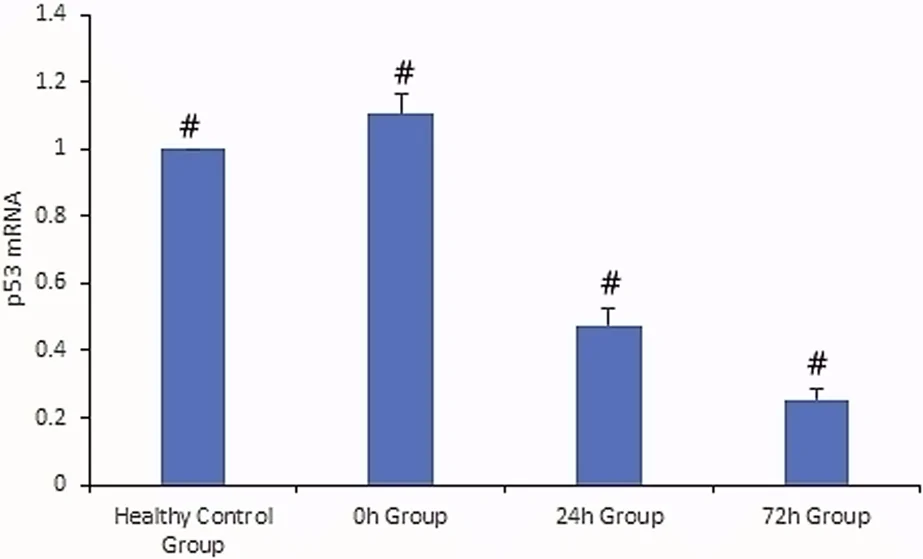
Changes in the expression of the p53 signaling pathway. The p53 signaling pathway was slightly upregulated immediately after puncture, followed by downregulation and upregulation at 24 and 72 h after puncture, respectively (P < 0.05).

Real-time polymerase chain reaction showed that p53 mRNA level was slightly upregulated immediately after puncture. However, from 24 h to 72 h after puncture, the pathway was downregulated (P < 0.05) (Figure 7).

### Changes in the expression of the JAK/STAT signaling pathway

**FIGURE 8:**
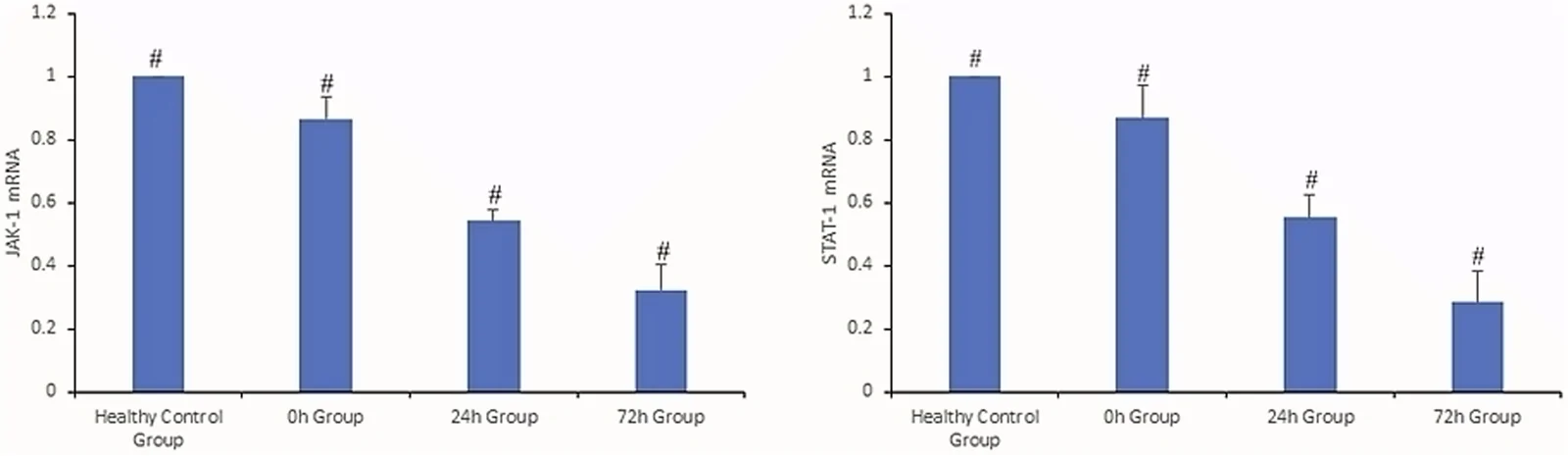
Changes in the expression of the JAK/STAT signaling pathway. The JAK/STAT signal pathway was downregulated from immediately after puncture until 72 h (all P < 0.05). Change of JAK mRNA was represented as **(a)**, Change of STAT mRNA was represented as **(b)**.

Real-time polymerase chain reaction has also shown that JAK-1 and STAT-1 mRNA levels were altered during acute stress. The JAK/STAT signal pathway was downregulated from immediately after puncture until 72 h (all P < 0.05) (Figure 8).

### Changes in the expression of the NF-κB signaling pathway

**FIGURE 9:**
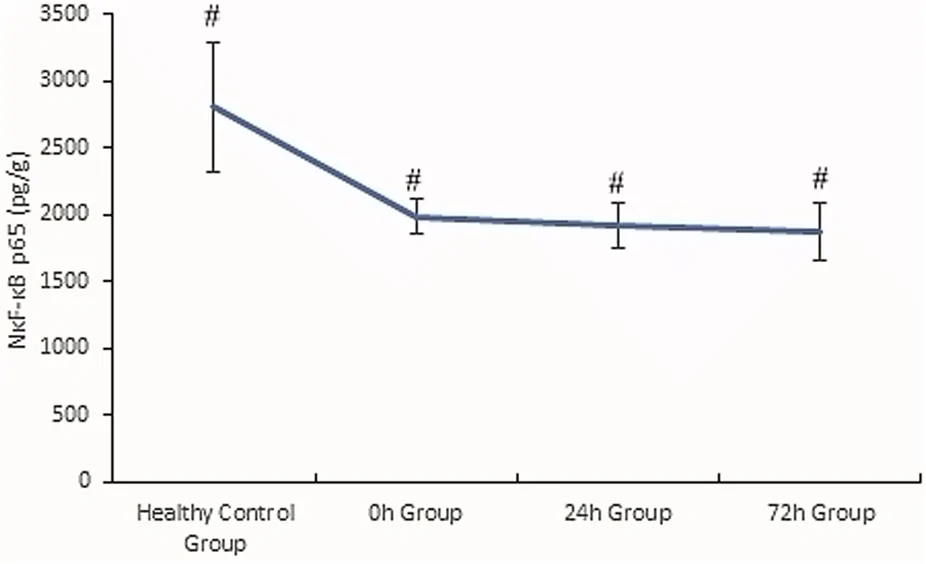
Changes in the expression of the NF-κB signaling pathway. The NF-κB signal pathway was downregulated from immediately after puncture until 72 h (P < 0.05).

ELISA showed that the NF-κB signaling pathway was altered during acute stress. The NF-κB signal pathway was downregulated from immediately after puncture until 72 h (all P < 0.05) (Figure 9).

### Changes in the expression of gastric inflammatory factors

**FIGURE 10:**
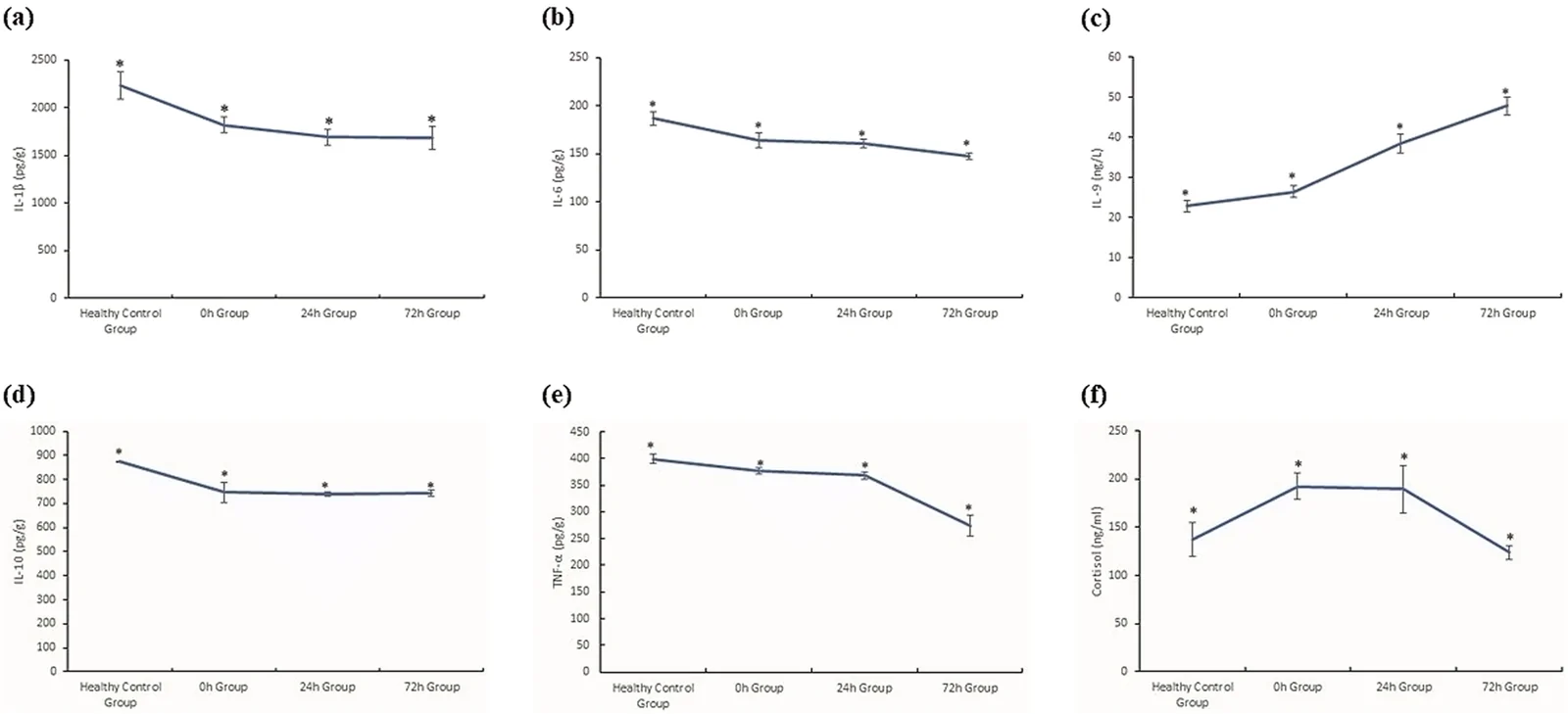
Changes in the expression of gastric inflammatory factors. IL-1β **(a)**, IL-6 **(b)**, IL-9 **(c)**, IL-10 **(d)**, TNF-α **(e)** and cortisol **(f)**, exhibited altered expression under acute stress. After puncture, IL-1β, IL-6, IL-10, and TNF-α were downregulated, IL-9 and cortisol showed an increased expression (all P< 0.05).

Furthermore, ELISA indicated that gastric inflammatory factors, including IL-1β, IL-6, IL-9, IL-10, and TNF-α, exhibited altered expression under acute stress. After puncture, IL-1β, IL-6, IL-10, and TNF-α were downregulated; however, IL-9 showed an increased expression (all P < 0.05) (Figure 10).

### Changes in the expression of gastric cortisol

**FIGURE 11:**
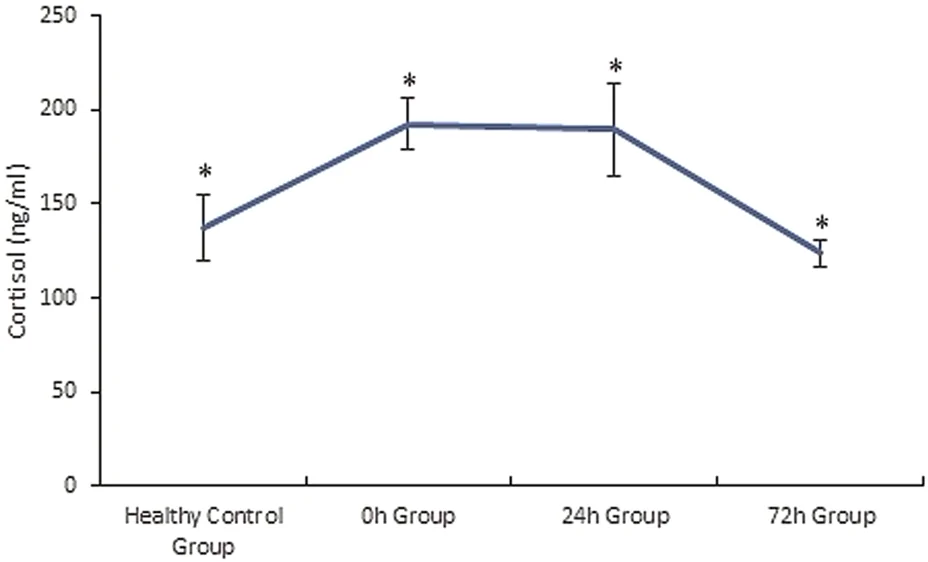
Changes in the expression of gastric cortisol. Cortisol showed an increased expression under acute stress (P< 0.05).

ELISA demonstrated that the expression of gastric cortisol was altered during acute stress. Gastric cortisol expression was increased immediately after puncture (all P < 0.05) (Figure 11).

### Changes in ROS and MDA expression of gastric cortisol

**FIGURE 12:**
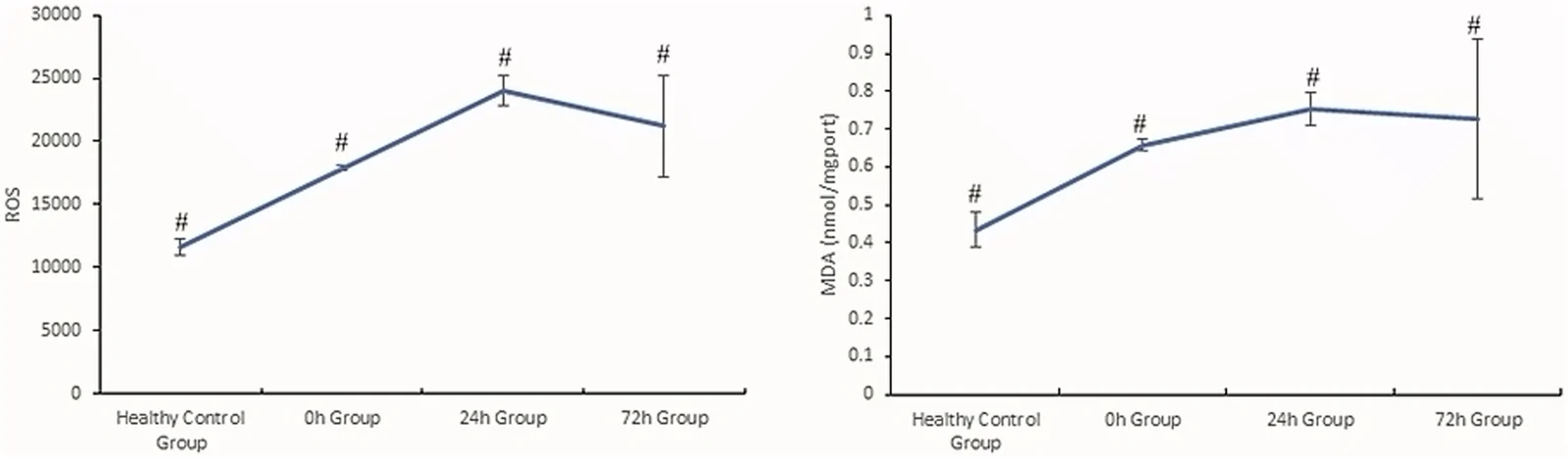
Changes in ROS and MDA expression of gastric tissue. Gastric ROS **(a)** and MDA **(b)** levels were increased from immediately after puncture to 24 h; however, ROS and MDA were gradually downregulated from 24 to 72 h after puncture (all P< 0.05).

Biochemical detection assays revealed that the levels of gastric ROS and MDA were altered during acute stress. Gastric ROS and MDA levels were increased from immediately after puncture to 24 h; however, ROS and MDA were gradually downregulated from 24 to 72 h after puncture (all P < 0.05) (Figure 12).

**FIGURE 13:**
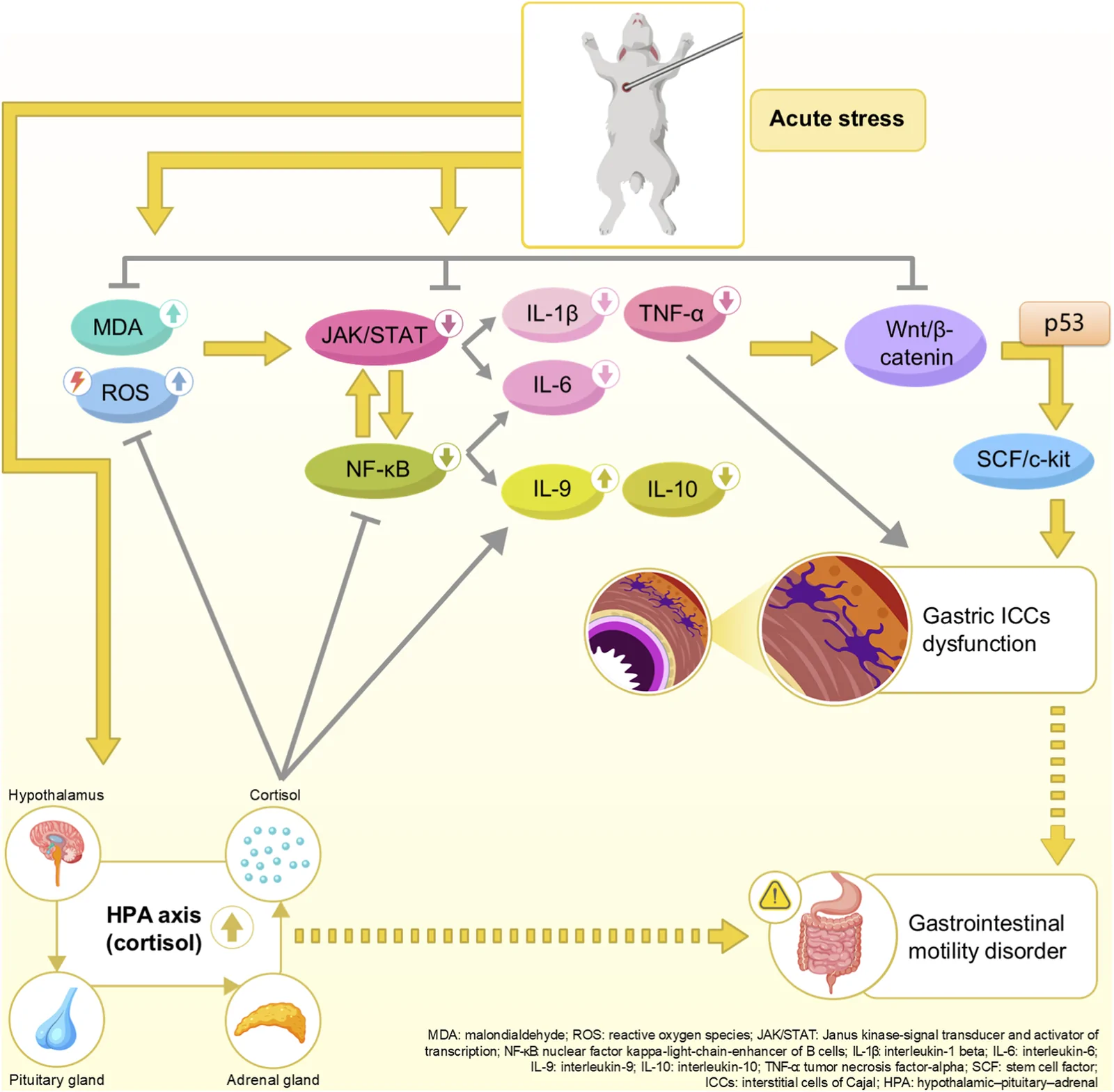
Mechanism of changes in gastric ICCs in the acute stress. Acute stress condition injures gastric ICCs through Wnt/β-catenin, p53, JAK/STAT, and NF-κB signaling pathways, as well as via SCF/c-kit pathway-mediated abnormalities and oxidative stress.

The potential cellular and molecular mechanism of changes in gastric ICCs in the acute stress condition is shown in Figure 13.

## Discussion

Traffic accidents are a common cause of thoracic injury, and thoracic trauma is a major contributor to trauma-related deaths globally (Benhamed et al., 2022). Trauma involves acute psychological and physical stress, which can lead to gastrointestinal dysmotility and exacerbate gastrointestinal symptoms (Tsukamoto et al., 2011; Hagos et al., 2024). Stress, including both acute and chronic forms, may induce gastrointestinal dysmotility by disrupting interactions between the gastrointestinal tract and the central and peripheral nervous systems (Drossman, 2016; Maselli et al., 2021).

ICCs are in the gastrointestinal tract and function as the pacemakers of gut motility, causing their involvement in motility disorders (Friedmacher and Rolle, 2023). ICCs express specific markers CD117/c-kit and CD34 (Ricci et al., 2024; Wang Q. et al., 2021). In addition, ICCs undergo apoptosis over time, and their regeneration is required for the maintenance of normal ICC networks in healthy gastrointestinal tissues (Gibbons et al., 2009). Gastric motility involves various regulatory mechanisms, such as gastric smooth muscle and nervous circuit activity, which are modulated by the ICCs in the gastric subepithelial connective tissue layer and smooth muscle. Gastric ICCs are injured and/or exhibit proliferation and growth disorders in gastrointestinal dysmotility, and abnormalities in motor activity result in impaired regional transit and symptoms (Huizinga et al., 2009). In addition to regulating gastric motility, ICCs mediate neurotransmission from enteric motor neurons (Chaudhury, 2016).

The tyrosine kinase receptor c-kit and its ligand SCF markedly influence the normal development, maturation, and phenotypic maintenance of gastric ICCs (Rich et al., 2003). Kit receptors also modulate the generation and rhythmicity of electrical activity to regulate the excitability of smooth muscle cells (Drumm et al., 2020). A defective SCF/c-kit pathway significantly influences the population and apoptosis of ICCs, functional changes in ICCs, and gastrointestinal motility diseases (Lennartsson and Rönnstrand, 2012; Li et al., 2024; Huang et al., 2020).

The Wnt/β-catenin signaling pathway is crucial in the regulation of various cellular processes, such as cell proliferation, differentiation, and migration (Liu et al., 2022). The aberrant activation or inhibition of the Wnt/β-catenin signaling pathway is closely associated with various diseases, including gastrointestinal motility disorders (Perugorria et al., 2019; Hayashi et al., 2021). In an acute stress condition, the Wnt ligand binds to the cell membrane receptor, activating the pathway, inhibiting the degradation of β-Catenin, and initiating the expression of downstream target genes and DNA damage (Hayashi et al., 2021). The impaired activities of the Wnt/β-catenin signaling pathway, anti-inflammation and mediation of the protein 53, cause disordered SCF/c-kit pathway and then impaired growth of the ICC stem cell, as well as altered cell proliferation and self-renewal, during acute stress (Hayashi et al., 2021; Huang et al., 2024).

The JAK/STAT and NF-κB signaling pathway is an intracellular signal transduction pathway and participates in crucial biological processes, such as immune cell proliferation, differentiation, and apoptosis. Cytokines, including IL-1, IL-6, IL-9, IL-10, and TNF-α, play a critical role in JAK/STAT and NF-κB signal transduction (Xin et al., 2020; Samra et al., 2025). Meanwhile, during acute stress, the hypothalamic-pituitary-adrenal axis can respond within minutes, leading to rapid and substantial secretion of glucocorticoids such as cortisol. As it’s only an indirect injury on stomach, the damage was relatively minor, proinflammatory factors including IL-1, IL-6, and TNF-α, which have act in concert and inflammatory responses are subsequently suppressed by central and neuroendocrine actions (Palazzolo and Quadri, 1990; Shalaby et al., 1989). In addition, inflammation-related signaling pathways, including the JAK/STAT and NF-κB pathways, are also inhibited (Di Bona et al., 2006; Schreck et al., 1992). In this study, after puncture, the Wnt/β-catenin signaling pathway was activated gradually, gastric IL-1, IL-6, IL-10, and TNF-α were decreased, IL-9 was increased, and the JAK/STAT and NF-κB signaling pathway was downregulated. IL-9 promotes ICC growth, maintains ICC health, and facilitates ICC repair (Ye et al., 2006; Huang et al., 2016).

ROS overproduction results in oxidative damage, causing cell death. Furthermore, ROS acts as a second messenger for kappa B (NF-κB) activation and blocking the JAK-STAT pathway (Di Bona et al., 2006; Ming et al., 2022; Trachootham et al., 2009). Moreover, oxidative stress induces gastric mucosal injury and may cause gastric motility dysfunction (López-Pingarrón et al., 2023). ROS-induced damage to the network-like structure formed by ICCs causes gastric dysmotility and modulates gene expression through the functional alterations of NF-κB (Jin et al., 2013). The Wnt/β-catenin signaling pathway suppresses ROS production and reduces NF-κB activity. Moreover, ROS affects inflammasomes in cells, causing altered production of TNF-α, IL-1, and IL-6 (van den Berg et al., 2001; Fang et al., 2024). In this study, ROS and MDA levels were increased immediately after puncture and decreased after acute stress was abrogated. Hence, ROS possibly drive gastric ICC disorder via the Wnt/β-catenin signaling pathway.

In the acute stress condition, the hypothalamic-pituitary-adrenal axis was activated, and cortisol was upregulated. These events exerted a significant anti-inflammatory effect, promoting IL-9 secretion. Furthermore, cortisol induces ROS production via a non-genomic glucocorticoid receptor-mediated pathway; thus, it promotes gastric ICC disorder (Qureshi et al., 2009; Huang et al., 2022; Espinoza et al., 2017).

This study has some limitations. First, there are antioxidant enzymes such as SOD or catalase, besides, direct gastric motility assessment all were not assessed. Second, the influence of gender and pathway inhibitors or activators for pathway change during acute stress process has also not been explored in this study. Finally, the functional changes in gastric ICCs still need to be explored in future studies.

Our study indicated that acute stress conditions injure gastric ICCs and may subsequently affect the function of gastric ICCs, contributing to functional gastrointestinal disorders.

## Data Availability

The original contributions presented in the study are included in the article/supplementary material, further inquiries can be directed to the corresponding author.
